# Comprehensive Comparison of Seven SARS-CoV-2-Specific Surrogate Virus Neutralization and Anti-Spike IgG Antibody Assays Using a Live-Virus Neutralization Assay as a Reference

**DOI:** 10.1128/spectrum.02314-22

**Published:** 2023-01-09

**Authors:** Marianne Graninger, Claudia Maria Jani, Elisabeth Reuberger, Katja Prüger, Philipp Gaspar, David Niklas Springer, Christian Borsodi, Lisa Weidner, Susanne Rabady, Elisabeth Puchhammer-Stöckl, Christof Jungbauer, Eva Höltl, Judith Helene Aberle, Karin Stiasny, Lukas Weseslindtner

**Affiliations:** a Center for Virology, Medical University of Vienna, Vienna, Austria; b Austrian Red Cross, Blood Service for Vienna, Lower Austria, and Burgenland, Vienna, Austria; c Karl Landsteiner University of Health Sciences, Department of General Health Studies, Division General and Family Medicine, Krems, Austria; d Center for Public Health, Medical University of Vienna, Vienna, Austria; Hôpital Saint-Louis

**Keywords:** SARS-CoV-2, antibodies, neutralizing, surrogate, neutralization, assay, immunoassays, microarray

## Abstract

Neutralizing antibodies (nAbs) are considered a valuable marker for measuring humoral immunity against SARS-CoV-2. However, live-virus neutralization tests (NTs) require high-biosafety-level laboratories and are time-consuming. Therefore, surrogate virus neutralization tests (sVNTs) have been widely applied, but unlike most anti-spike (S) antibody assays, NTs and sVNTs are not harmonized, requiring further evaluation and comparative analyses. This study compared seven commercial sVNTs and anti-S-antibody assays with a live-virus NT as a reference, using a panel of 720 single and longitudinal serum samples from 666 convalescent patients after SARS-CoV-2 infection. The sensitivity of these assays for detecting antibodies ranged from 48 to 94% after PCR-confirmed infection and from 56% to 100% relative to positivity in the in-house live-virus NT. Furthermore, we performed receiver operating characteristic (ROC) curve analyses to determine which immunoassays were most suitable for assessing nAb titers exceeding a specific cutoff (NT titer, ≥80) and found that the NeutraLISA and the cPass assays reached the highest area under the curve (AUC), exceeding 0.91. In addition, when the assays were compared for their correlation with nAb kinetics over time in a set of longitudinal samples, the extent of the measured decrease of nAbs after infection varied widely among the evaluated immunoassays. Finally, in vaccinated convalescent patients, high titers of nAbs exceeded the upper limit of the evaluated assays’ quantification ranges. Based on data from this study, we conclude that commercial immunoassays are acceptable substitutes for live-virus NTs, particularly when additional adapted cutoffs are employed to detect nAbs beyond a specific threshold titer.

**IMPORTANCE** While the measurement of neutralizing antibodies is considered a valuable tool in assessing protection against SARS-CoV-2, neutralization tests employ live-virus isolates and cell culture, requiring advanced laboratory biosafety levels. Including a large sample panel (over 700 samples), this study provides adapted cutoff values calculated for seven commercial immunoassays (including four surrogate neutralization assays and a protein-based microarray) that robustly correlate with specific titers of neutralizing antibodies.

## INTRODUCTION

Neutralizing antibodies (nAbs) against severe acute respiratory syndrome coronavirus 2 (SARS-CoV-2) are mainly directed against the receptor-binding domain (RBD) of the viral spike (S) protein, thus inhibiting virus entry into the host cell, and are, in addition to cell-mediated immunity, a critical factor for protection against (re)infection (1–6). Therefore, live-virus neutralization tests (NTs) are essential for quantitatively measuring SARS-CoV-2-specific humoral immunity.

However, the performance of these assays requires advanced biosafety levels in specialized laboratories. Furthermore, NTs are usually in-house tests employing live-virus isolates and cell culture and are, therefore, difficult to standardize. In addition, these assays are time-consuming and labor-intensive and are of only limited feasibility for laboratory routine diagnostics. Thus, commercial enzyme-linked immunosorbent assays (ELISAs) and chemiluminescent immunoassays (CLIAs) have been widely distributed as a more convenient and easy-to-standardize alternative for measuring antibody binding, albeit without directly assessing functional capabilities (7, 8). In addition, surrogate virus neutralization tests (sVNTs) have been developed that quantify antibody-mediated inhibition of the binding between the RBD and its cellular receptor, the angiotensin-converting enzyme 2 (ACE2), using the common principle of ELISA (9).

While the most common commercial ELISAs and CLIAs use binding antibody units (BAU) per milliliter, the standardized unit proposed by the World Health Organization, commercial sVNTs have not been harmonized (10). Comprehensive comparative evaluations of these assays are thus still necessary, optimally including as many sVNTs as possible and using large serum panels to determine which assays are the best substitutes for live-virus NTs in different applications (e.g., detecting low levels of nAbs versus identifying nAb titers exceeding a specific cutoff).

Previous studies correlated different combinations of commercial ELISAs, CLIAs, and sVNTs with live-virus NTs, but only a few included more than two sVNTs while at the same time using a sufficiently large sample panel (7, 11–33). This study performed a comprehensive comparative evaluation and included 720 single and longitudinal samples from 666 convalescent patients after SARS-CoV-2 infection and seven commercial immunoassays, including four sVNTs and one protein-microarray assay, using an in-house live-virus NT as a reference.

While their sensitivities ranged from 48% to 100%, the evaluated assays displayed similar abilities to detect neutralizing antibodies with a live-virus NT titer of ≥80 (all areas under the receiver operating characteristic [ROC] curve [AUC] > 0.85). In addition, our data indicate additional cutoff values with statistical significance for measuring nAb levels exceeding this specific NT titer. Notably, the measurement of decreasing nAb levels assessed over time strongly depended on the assay used.

## RESULTS

### Characteristics of convalescent individuals after SARS-CoV-2 infection.

A total of 720 serum samples from 666 convalescent individuals after SARS-CoV-2 infection were analyzed with seven commercial SARS-CoV-2-specific antibody assays using a live-virus NT as a reference. Table 1 shows the demographic data for these individuals, the interval between SARS-CoV-2 infection and vaccination, and the time point of blood sampling.

**TABLE 1:** Demographic data of COVID-19-convalescent individuals and controls

Group	No. of individuals	Median age (yrs) (range)	No. (%) of F/M/unknown sex	No. of samples	Median (range) day of sampling^*a*^		
					DPO^*b*^	DPO or DPV	DPV
Whole cohort	666	45 (14–97)	301/359/6 (45/54/1)	720	82 (14–385)		
PCR-confirmed patients	603	44 (15–97)	265/332/6 (44/55/1)	657	78 (14–385)		
Convalescent blood donors	155	49 (20–69)	48/101/6 (32/68/2)	155	49 (32–163)		
Convalescent patients with follow-up	34	26 (20–57)	14/20/0 (41/59/0)	68	34 (14–89)^*c*^	177 (90–212)^*d*^	
Vaccinated convalescent patients^*e*^	20	33 (23–74)	14/6/0 (70/30/0)	40^*f*^	37 (19–330)^*g*^	27 (12–63)^*h*^	73 (55–91)^*i*^
Serologically confirmed patients	63	47 (14–78)	36/27/0 (57/43/0)	63	181 (34–263)		
Controls	234	45 (14–91)	112/122/0 (48/52/0)	234			

a DPO, days post-symptom onset; DPV, days postvaccination.

b Day of symptom onset was available for 501/666 (75%) individuals.

c Initial sample.

d Follow-up sample (DPO).

e Individuals received the BioNTech Pfizer (*n* = 17), Johnson & Johnson (*n* = 2), or Astra Zeneca (*n* = 1) vaccine.

f Including one pre and one postvaccination sample per patient.

g Prevaccination sample.

h Sample after the first vaccination (*n* = 18) (DPV).

i Sample after the second vaccination (*n* = 18).

In 603 individuals, SARS-CoV-2 infection was verified by RT-PCR from nasopharyngeal swabs, which occurred between March 2020 and January 2021. This was the first positive RT-PCR result obtained from all individuals, indicating primary SARS-CoV-2 infection. Of the 603 individuals, 155 were volunteer convalescent plasma donors at the Austrian Red Cross, and this subgroup was designated “convalescent plasma donors” (*n* = 155). The other RT-PCR-confirmed convalescent individuals were designated “PCR confirmed” (*n* = 448). For 63 individuals, there was no documentation of RT-PCR-positivity, but SARS-CoV-2 infection was verified by in-house live-virus NT positivity (“serologically confirmed”).

Data on clinical symptoms of SARS-CoV-2 infection were available for 513 convalescent individuals. The most common symptoms were fever (*n* = 278 [54.2%]), cough (*n* = 240 [46.8%]), dyspnea (*n* = 193 [45.3%]) and fatigue (*n* = 81 [15.8%]). Thirty convalescent individuals (5.8%) reported an asymptomatic course. None of the convalescent individuals with available clinical data was hospitalized. The date of symptom onset was available for 501 of 666 individuals (75%), and serum samples were obtained after a median of 82 days (range, 14 to 385) after the onset of symptoms.

### Sensitivity and specificity of the SARS-CoV-2-specific antibody assays.

First, the sensitivity to detect the lowest levels of any SARS-CoV-2-specific antibodies and serologically verify infection was calculated for all seven commercial assays and the in-house live-virus NT using samples from PCR-confirmed cases (*n* = 603; true positives defined by RT-PCR positivity). The results are summarized in Table 2. The sensitivities to detect SARS-CoV-2-specific antibodies and identify past infection varied widely among the evaluated antibody assays and ranged from 93.86% (SARS-CoV-2 ViraChip IgG assay, RBD IgG [Viramed]) to 47.76% (SARS-CoV-2-NeutraLISA [Euroimmun]). Our live-virus NT showed a sensitivity of 83.58% for PCR-confirmed convalescent patients. Table 2 also shows the detection rates in a subgroup of samples from individuals in whom SARS-CoV-2 infection was verified only serologically by the in-house live-virus NT because no RT-PCR result was available (*n* = 63).

**TABLE 2:** Sensitivities of the evaluated immunoassays^*a*^

Test	Parameter	PCR confirmed (*n* = 603)				Serologically confirmed (*n* = 63)				NT positive (*n* = 567)				All samples (*n* = 666)			
		% sensitivity (95% CI)	No.	% PPV (95% CI)	% NPV (95% CI)	% sensitivity (95% CI)	No.	% PPV (95% CI)	% NPV (95% CI)	% sensitivity (95% CI)	No.	% PPV (95% CI)	% NPV (95% CI)	% sensitivity (95% CI)	No.	% PPV (95% CI)	% NPV (95% CI)
Neutralization assay	Positive (>1:10)	83.58 (80.41–86.32)	504	100.00 (99.24–100.00)	70.27 (65.15–74.93)	100.00 (94.25–100.00)	63	100.00 (94.25–100.00)	100.00 (98.38–100.00)	100.00 (99.33–100.00)	567	100.00 (99.33–100.00)	100.00 (98.38–100.00)	85.14 (82.23–87.64)	567	100.00 (99.33–100.00)	70.27 (65.15–74.93)
cPass SARS-CoV-2 neutralization antibody detection kit (GenScript)	Positive (≥30% IH)	78.94 (75.51–82.00)	476	100.00 (99.20–100.00)	64.82 (59.76–69.57)	93.65 (84.78–97.50)	59	100.00 (93.89–100.00)	98.32 (95.76–99.34)	91.36 (88.76–93.40)	518	100.00 (99.26–100.00)	82.69 (77.85–86.65)	80.33 (77.14–83.17)	535	100.00 (99.29–100.00)	64.11 (59.07–68.86)
SARS-CoV-2-NeutraLISA (Euroimmun)	Positive (≥35% IH)	47.76 (43.80–51.75)	288	98.29 (96.07–99.27)	42.10 (38.02–46.29)	50.79 (38.76–62.73)	32	86.49 (72.02–94.09)	88.08 (83.57–91.47)	56.44 (52.33–60.46)	320	98.46 (96.45–99.34)	48.11 (43.65–52.59)	48.05 (44.28–51.84)	320	98.46 (96.45–99.34)	39.83 (35.91–43.88)
	Borderline (≥20% IH)	66.00 (62.13–69.67)	398	98.76 (97.13–99.47)	52.76 (48.06–57.42)	63.49 (51.15–74.28)	40	88.89 (76.50–95.16)	90.87 (86.68–93.84)	76.01 (72.33–79.35)	431	98.85 (97.34–99.51)	62.74 (57.67–67.54)	65.77 (62.08–69.27)	438	98.87 (97.39–99.52)	50.11 (45.54–54.67)
ACE2-RBD neutralization assay (DiaPro)	Positive (>1 Co/S)	85.74 (82.72–88.30)	517	100.00 (99.26–100.00)	73.13 (68.01–77.69)	96.83 (89.14–99.44)	61	100.00 (94.08–100.00)	99.15 (96.96–99.85)	95.77 (93.78–97.14)	543	100.00 (99.30–100.00)	90.70 (86.53–93.67)	86.79 (84.00–89.15)	578	100.00 (99.34–100.00)	72.67 (67.56–77.25)
SARS-CoV-2-AK Surrogate neutralization test (TECOmedical)	Positive (≥20% IH)	79.93 (76.55–82.94)	482	100.00 (99.21–100.00)	65.92 (60.84–70.65)	95.24 (86.91–98.70)	60	100.00 (93.98–100.00)	98.73 (96.35–99.65)	91.53 (88.95–93.56)	519	100.00 (99.27–100.00)	82.98 (78.16–86.91)	81.38 (78.25–84.15)	542	100.00 (99.33–100.00)	65.36 (60.29–70.11)
Anti-SARS-CoV-2-QuantiVac-ELISA (IgG) (Euroimmun)	Positive (≥35.2 BAU/mL)	74.46 (70.83–77.78)	449	99.56 (98.40–99.92)	60.10 (55.14–64.87)	90.48 (80.74–95.56)	57	96.61 (88.46–99.40)	97.48 (94.61–98.84)	87.30 (84.31–89.79)	495	99.60 (98.54–99.93)	76.32 (71.23–80.75)	75.98 (72.59–79.07)	506	99.61 (98.58–99.93)	59.18 (54.25–63.94)
	Borderline (≥25.6 BAU/mL)	81.76 (78.48–84.64)	493	99.60 (98.54–99.93)	67.84 (62.71–72.57)	95.24 (86.91–98.70)	60	96.77 (88.98–99.43)	98.72 (96.31–99.65)	94.00 (91.74–95.68)	533	99.63 (98.65–99.93)	87.22 (82.67–90.71)	83.03 (79.99–85.69)	553	99.64 (98.70–99.94)	67.25 (62.13–71.99)
LIAISON SARS-CoV-2 TrimericS IgG (DiaSorin)	Positive (≥33.8 BAU/mL)	82.42 (79.18–85.25)	497	100.00 (99.23–100.00)	68.82 (63.71–73.51)	90.48 (80.74–95.56)	57	100.00 (93.69–100.00)	97.50 (94.65–98.85)	92.42 (89.94–94.32)	524	100.00 (99.27–100.00)	84.48 (79.74–88.27)	83.18 (80.15–85.83)	554	100.00 (99.33–100.00)	67.63 (62.53–72.34)
SARS-CoV-2 ViraChip IgG assay (Viramed)																	
RBD IgG	Positive (≥15 BAU/mL)	93.86 (91.66–95.52)	566	99.30 (98.21–99.73)	86.14 (81.48–89.78)	100.00 (94.25–100.00)	63	94.03 (85.63–97.65)	100.0 (98.36–100.00)	99.47 (98.46–99.86)	564	99.30 (98.20–99.73)	98.71 (96.28–99.65)	94.44 (92.44–95.94)	629	99.37 (98.39–99.75)	86.14 (81.48–89.78)
S1 IgG	Positive (≥25 BAU/mL)	88.06 (85.23–90.41)	531	99.81 (98.94–99.99)	76.39 (71.32–80.81)	98.41 (91.54–99.92)	62	98.41 (91.54–99.92)	99.57 (97.62–99.98)	96.30 (94.40–97.56)	546	99.82 (98.97–99.99)	91.73 (87.69–94.53)	89.04 (86.44–91.19)	593	99.83 (99.05–99.99)	76.14 (71.06–80.58)

a CI, confidence interval; PPV, positive predictive value; NPV, negative predictive value. PPV and NPV were calculated based on the test specificities as shown in Table S3.

Then, positivity in the in-house live-virus NT was used as a reference to assess the assays’ sensitivity to detect any level of nAbs (*n* = 567; true positives defined by positivity in the live-virus NT). Here, the sensitivities of the commercial immunoassays ranged from 99.47% (SARS-CoV-2 ViraChip IgG assay, RBD IgG [Viramed]) to 56.44% (SARS-CoV-2-NeutraLISA [Euroimmun]). Sensitivity calculation was performed with samples from all individuals, including prevaccination samples from vaccinees (*n* = 20).

The specificity of all seven SARS-CoV-specific antibody assays was assessed with prepandemic samples from 234 healthy individuals, tested at the Center for Virology for measles virus-, mumps virus- and rubella virus-specific antibodies after vaccination. The specificity results are shown in Table S2 in the supplemental material. Overall, the assays’ specificities were very high, ranging from 100% to 97.86%.

Antibodies against the seasonal human coronaviruses (HCoV) 229E, OC43, NL63, and HKU1 were assessed in SARS-CoV-2-infected individuals and prepandemic control samples using the SARS-CoV-2 ViraChip IgG microarray (Viramed Biotech, Planegg, Germany). Antibody positivity against at least one HCoV was 82.2% in SARS-CoV-2-infected individuals and did not differ from the positivity rate of 84.2% in prepandemic control samples (*P* = 0.551, Fisher’s exact test).

### Performance of commercial SARS-CoV-2-specific antibody assays in correlation with nAbs.

**FIG 1:**
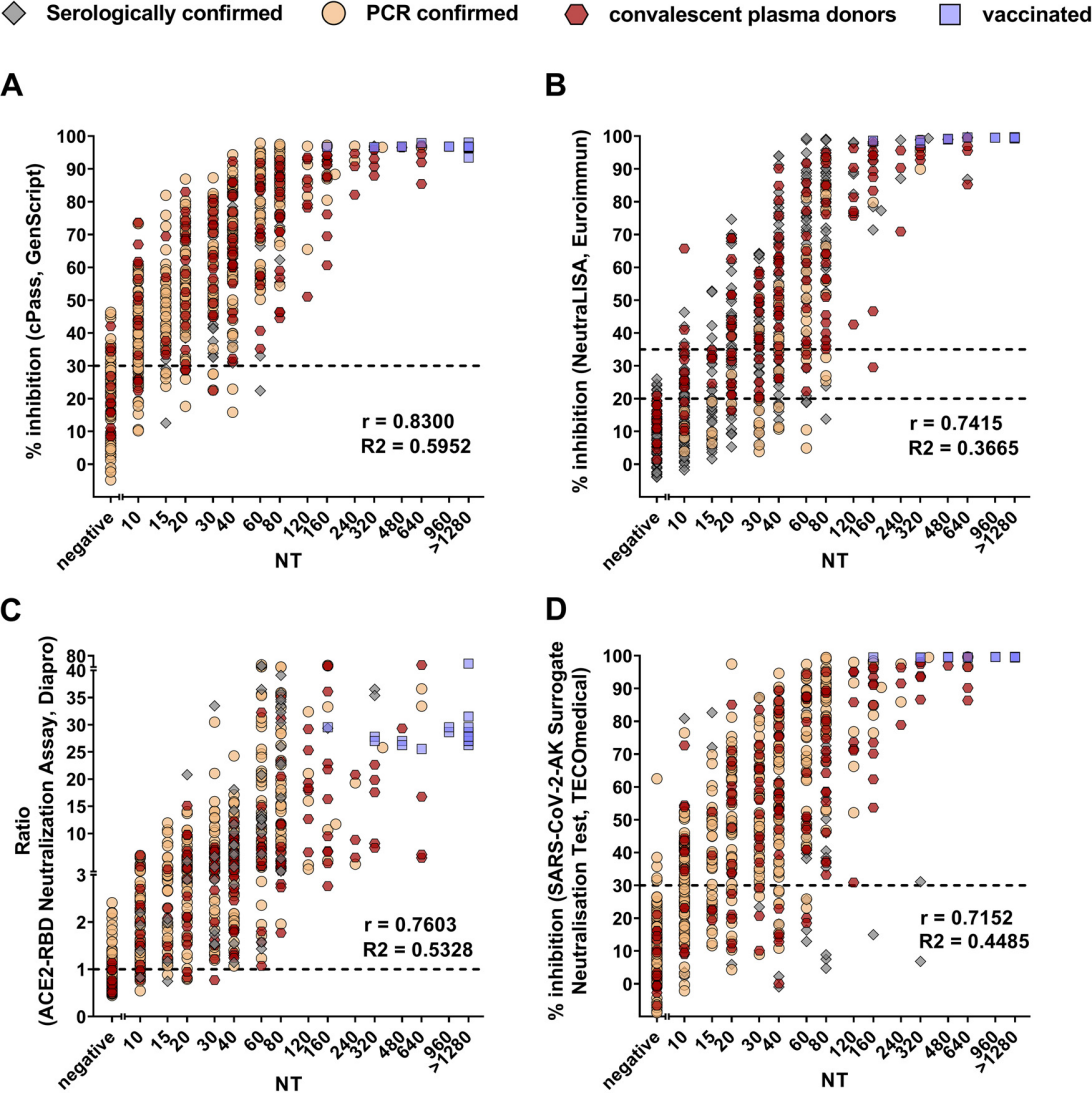
Correlation analyses of sVNTs and nAb titers measured by in-house live-virus NT. Spearman correlation coefficients (*r*) and coefficients of determination (*R*^2^) are indicated. (A) cPass (GenScript); (B) SARS-CoV-2-NeutraLISA (Euroimmun); (C) ACE2-RBD neutralization assay (DiaPro); (D) TECO SARS-CoV-2 neutralization antibody assay (TECOmedical). Symbols represent samples from different cohorts, as indicated at the top. The dotted lines indicate the cutoffs of the specific assays.

**FIG 2:**
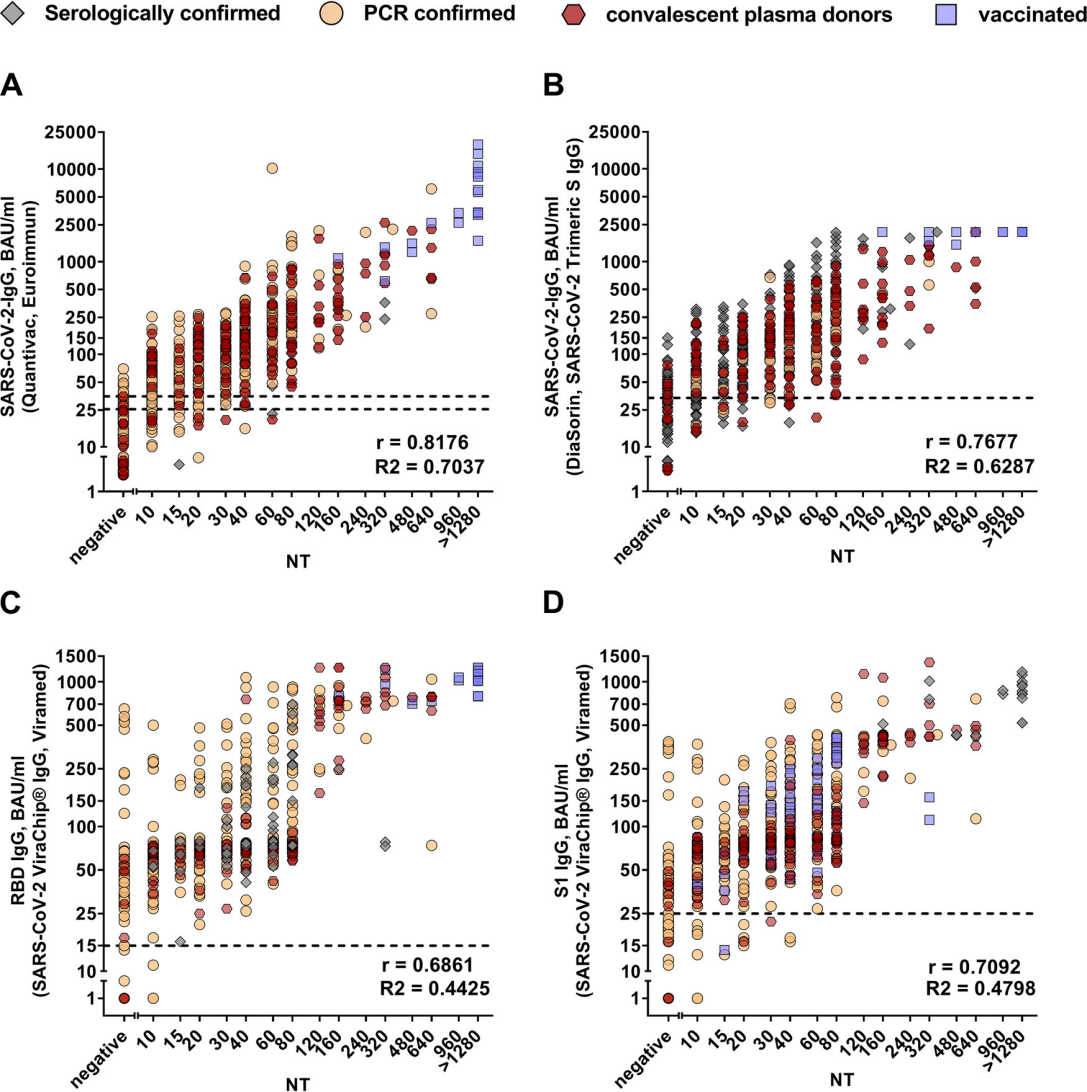
Correlation analyses of the IgG antibody binding tests and nAb titers measured with an in-house live-virus NT. Spearman correlation coefficients (*r*) and coefficients of determination (*R*^2^) are indicated. (A) Anti-SARS-CoV-2 QuantiVac ELISA (Euroimmun); (B) Liaison SARS-CoV-2 TrimericS IgG CLIA (DiaSorin); (C and D) SARS-CoV-2 ViraChip IgG microarray (Viramed Biotech) RBD (C) and S1-directed IgG (D). Symbols represent samples from different cohorts, as indicated at the top. The dotted lines indicate the cutoffs of the specific assays.

Next, the ability of the commercial SARS-CoV-2-specific antibody assays to quantify nAbs against SARS-CoV-2 was analyzed by correlating live-virus NT titers with the results from the sVNTs (as percent RBD-ACE2 inhibition or cutoff/sample ratio [Co/S]), the ELISA, the CLIA, and the microarray (in BAU per milliliter). As shown in Fig. 1 (sVNTs) and Fig. 2 (ELISA, CLIA, and microarray), there was a statistically significant correlation (*P* < 0.0001) between live-virus NT titers and quantitative results from all commercial antibody assays. However, the correlation strength varied among the tests, with values of *r* ranging from 0.83 for cPass (GenScript) to 0.69 for Anti-RBD-IgG of the ViraChip IgG assay (Fig. 1). In addition, the extent of the correlation differed within assays depending on the concentration range of nAbs (Fig. 1 and 2).

**FIG 3:**
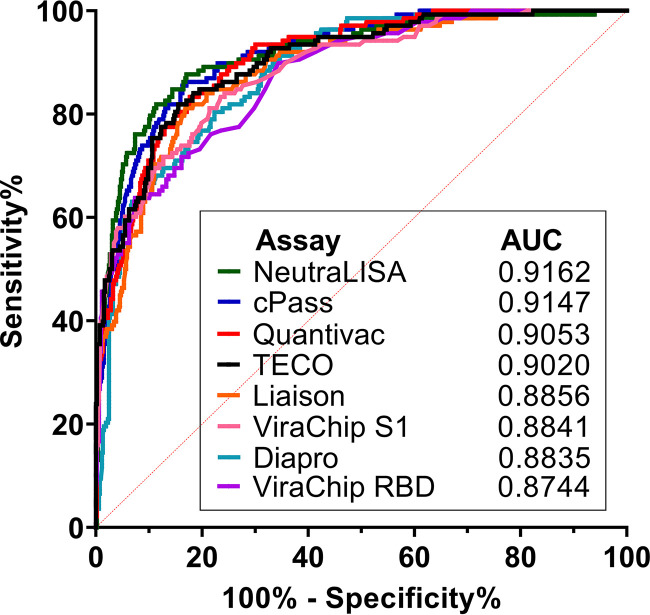
ROC curves of all tested antibody assays, calculated using an NT titer of ≥80 as the cutoff. The table shows the assays and associated AUC values.

Therefore, we then analyzed the performance of the commercial antibody assays to detect nAbs with “high-level” titers, as defined by previous observations in our laboratory, using ROC curve analyses. A nAb titer of ≥80 in the live-virus NT was defined as a cutoff. The results are shown in Fig. 3. The AUC for all evaluated antibody assays ranged from 0.92 for SARS-CoV-2-NeutraLISA (Euroimmun) to 0.87 for the ACE2-RBD neutralization assay (DiaPro). The four antibody assays with the highest AUC included three sVNTs: the SARS-CoV-2-NeutraLISA (Euroimmun) with an AUC of 0.92, cPass (GenScript) with an AUC of 0.92, and the TECO SARS-CoV-2 neutralization antibody assay (TECOmedical) with an AUC of 0.90.

Table 3 shows the specific cutoffs calculated using ROC analyses for the commercial antibody assays that would be optimal to detect a nAb titer of ≥80 with a specificity of >90%, >95%, and >99%. Furthermore, Table 3 includes the optimal cutoff determined by Youden’s index for the highest combination of sensitivity and specificity. Of note, the cutoffs providing a specificity of >95% to detect nAb titers of ≥80 yielded higher detection rates (sensitivity) for most of the sVNTs than for the ELISA, CLIA, and microarray, indicating a slightly higher diagnostic ability of the sVNTs to identify high nAb titers.

**TABLE 3:** Cutoffs for commercial immunoassays to detect nAb titers of ≥ 80 after SARS-CoV-2 infection^*a*^

Test	Cutoff	% (95% CI)			
		Sensitivity	Specificity	PPV	NPV
cPass SARS-CoV-2 neutralization antibody detection kit (GenScript)	82.09% IH^*b*^	74.64 (66.78–81.16)	90.15 (87.31–92.41)	66.45 (58.70–73.41)	93.715 (90.62–95.03)
	86.94% IH^*c*^	60.87 (52.54–68.61)	95.08 (92.88–96.62)	76.36 (67.62–83.33)	90.29 (87.54–92.48)
	94.43% IH^*d*^	28.99 (22.07–37.04)	99.05 (97.80–99.59)	88.89 (76.50–95.16)	84.22 (81.14–86.87)
	75.22% IH^*e*^	85.51 (78.67–90.42)	83.71 (80.32–86.62)	57.84 (50.98–64.41)	95.67 (93.41–97.18)

SARS-CoV-2-NeutraLISA (Euroimmun)	67.05% IH^*b*^	77.54 (69.88–3.70)	90.15 (87.31–92.41)	67.30 (59.67–74.10)	93.89 (91.45–95.66)
	77.26% IH^*c*^	68.84 (60.69–75.97)	95.08 (92.88–96.62)	78.51 (70.38–8.89)	92.11 (89.54–94.09)
	93.98% IH^*d*^	35.51 (28.01–43.78)	99.05 (97.80–99.59)	90.74 (80.09–95.98)	85.46 (82.44–88.03)
	55.02% IH^*e*^	87.68 (81.16–92.16)	82.95 (79.51–85.92)	57.35 (50.60–63.83)	96.26 (94.10–97.65)

ACE2-RBD neutralization assay (DiaPro)	12.63 Co/S ratio^*b*^	64.49 (56.22–71.99)	90.15 (87.31–92.41)	63.12 (54.91–70.64)	90.67 (87.87–92.87)
	18.17 Co/S ratio^*c*^	53.62 (45.32–61.73)	95.08 (92.88–96.62)	74.00 (64.63–81.60)	88.69 (85.82–91.04)
	35.49 Co/S ratio^*d*^	9.42 (5.59–15.45)	99.05 (97.80–99.59)	72.22 (49.13–87.50)	80.71 (77.49–83.56)
	6.51 Co/S ratio^*e*^	80.43 (73.03–86.19)	77.65 (73.91–81.00)	48.47 (42.08–54.92)	93.82 (91.16–95.72)

SARS-CoV-2-AK surrogate neutralization test (TECOmedical)	78.72% IH^*b*^	69.57 (61.44–76.63)	90.15 (87.31–92.41)	64.86 (56.89–72.09)	91.89 (89.22–93.95)
	86.78% IH^*c*^	54.35 (46.03–62.43)	95.08 (92.88–96.62)	74.26 (64.95–81.78)	88.85 (85.99–91.19)
	93.18% IH^*d*^	39.13 (31.39–47.46)	99.24 (98.07–99.71)	93.10 (83.57–97.29)	86.18 (83.21–88.70)
	70.00% IH^*e*^	81.88 (74.62–87.42)	84.47 (81.13–87.31)	57.95 (50.93–64.66)	94.69 (92.28–96.38)

Anti-SARS-CoV-2 QuantiVac (Euroimmun)	231.3 BAU/mL^*b*^	71.01 (62.69–77.93)	90.11 (87.27–92.38)	65.33 (57.42–72.48)	92.22 (89.58–94.23)
	315 BAU/mL^*c*^	51.45 (43.18–59.64)	95.06 (92.86–96.60)	73.20 (63.62–81.00)	88.18 (85.27–90.59)
	671 BAU/mL^*d*^	33.33 (26.01–41.56)	99.05 (97.79–99.59)	90.20 (79.02–95.74)	84.99 (81.95–87.60)
	174.5 BAU/mL^*e*^	83.33 (76.23–88.63)	82.32 (78.83–85.34)	55.29 (48.50–61.89)	94.96 (92.55–96.62)

SARS-CoV-2 TrimericS-IgG (DiaSorin)	327 BAU/mL^*b*^	63.77 (55.48–71.31)	90.15 (87.31–92.41)	62.86 (54.61–70.42)	90.49 (87.69–92.72)
	487 BAU/mL^*c*^	46.38 (38.27–54.68)	95.08 (92.88–96.62)	71.11 (61.04–79.46)	87.15 (84.17–89.64)
	875 BAU/mL^*d*^	34.06 (26.68–42.30)	99.05 (97.80–99.59)	90.38 (79.39–95.82)	85.18 (82.15–87.77)
	211 BAU/mL^*e*^	81.16 (73.83–86.81)	83.14 (79.71–86.10)	55.72 (48.81–62.42)	94.41 (91.93–96.16)

SARS-CoV-2 ViraChip RBD IgG (Viramed)	196 BAU/mL^*b*^	63.77 (55.48 –71.31)	90.13 (87.29–92.40)	62.86 (54.61–70.42)	90.48 (87.66–92.70)
	292.5 BAU/mL^*c*^	54.35 (46.03–62.43)	95.07 (92.87–96.61)	74.26 (64.95–81.78)	88.83 (85.96–91.17)
	652.5 BAU/mL^*d*^	40.58 (32.75–48.92)	99.05 (97.80–99.59)	91.80 (82.21–96.45)	86.42 (83.46–88.93)
	232.5 BAU/mL^*e*^	63.77 (55.48–71.31)	92.60 (90.04–94.54)	69.29 (60.80–76.65)	90.71 (87.96–92.88)

SARS-CoV-2 ViraChip S1 IgG (Viramed)	190.5 BAU/mL^*b*^	65.94 (57.7–73.32)	90.15 (87.31–92.41)	63.64 (55.49–71.07)	91.01 (88.25–93.17)
	287.5 BAU/mL^*c*^	57.97 (49.63–65.88)	95.45 (93.33–96.93)	76.92 (67.95–83.97)	89.68 (86.89–91.93)
	390 BAU/mL^*d*^	39.86 (32.07–48.19)	99.05 (97.80–99.59)	91.67 (81.93–96.39)	86.30 (83.34–88.81)
	102.5 BAU/mL^*e*^	83.33 (76.23–88.63)	76.52 (72.72–79.93)	48.12 (41.60–54.43)	94.61 (92.05–96.38)

a CI, confidence interval; PPV, positive predictive value; NPV, negative predictive value; IH, inhibition.

b Cutoff for a specificity of >90%.

c Cutoff for a specificity of >95%.

d Cutoff for a specificity of >99%.

e Cutoff determined by Youden’s index for the highest combination of sensitivity and specificity.

### Ability of antibody assays to assess nAbs over time in longitudinal samples.

**FIG 4:**
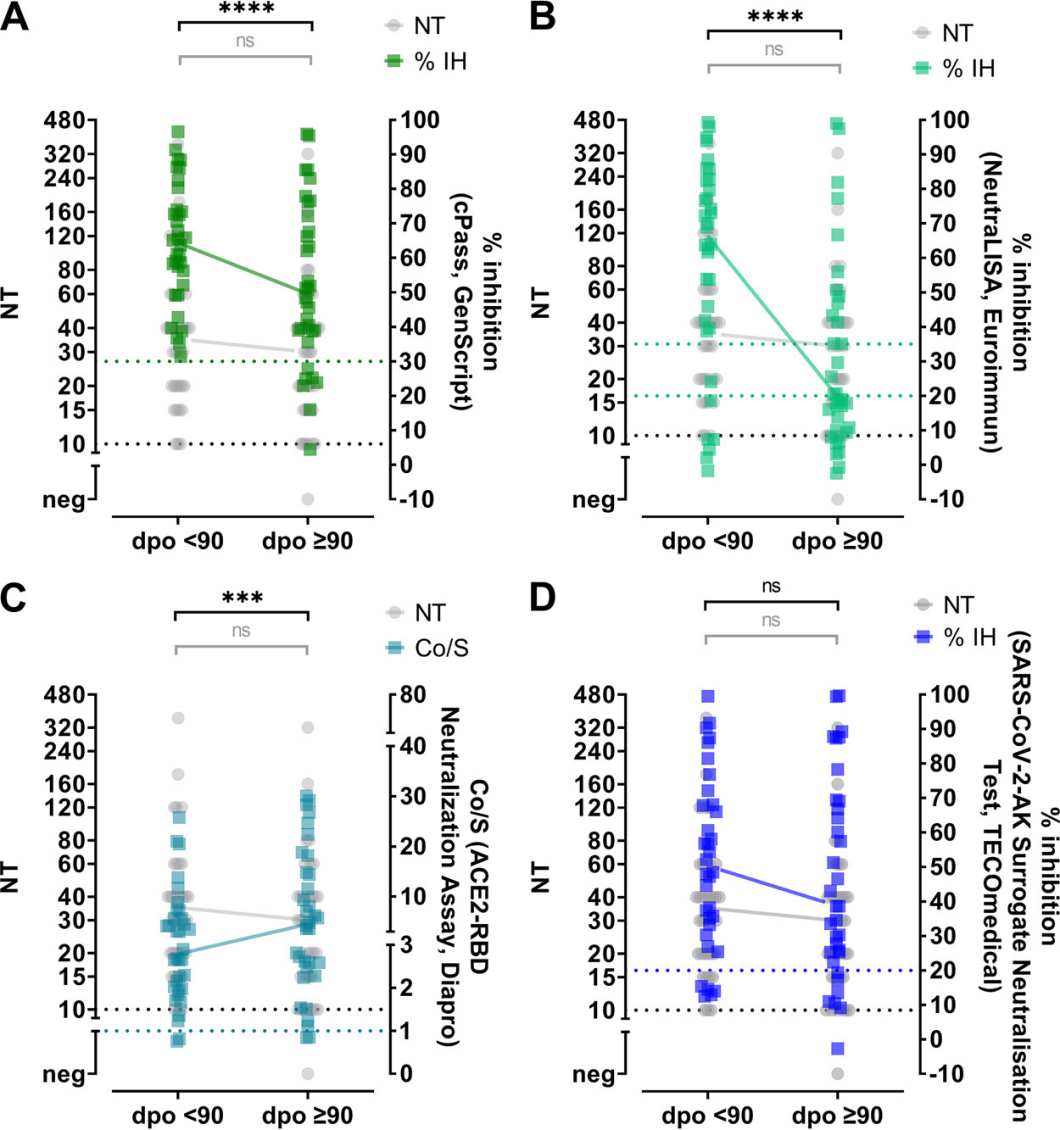
Longitudinal changes in neutralizing antibodies measured by live-virus NT and sVNTs. Samples from 34 convalescent individuals for whom the first sample was taken <90 days postonset (dpo) and an additional sample ≥90 days postonset were tested with an NT and all sVNTs. (A) cPass (GenScript); (B) SARS-CoV-2-NeutraLISA (Euroimmun); (C) ACE2-RBD neutralization assay (DiaPro); (D) TECO SARS-CoV-2 neutralization antibody assay (TECOmedical). The dotted lines indicate the cutoffs of the specific assays. Connected lines show medians. Comparison of paired values was performed by the Wilcoxon test. Gray brackets show the results for NT values, and black brackets show those for the respective commercial antibody assay. ***, *P* ≤ 0.001; ****, *P* ≤ 0.0001; ns, not significant. IH, inhibition.

Next, the reliability of commercial antibody assays was evaluated to quantify changes in nAb levels over time. To this end, serum samples from 34 convalescent individuals for whom follow-up samples were available were analyzed. The first sample was obtained within 90 days after symptom onset (after a median of 34 days; range, 14 to 89) and the follow-up sample at least 90 days postonset (after a median of 177 days; range, 90 to 212) (Table 1). The minimal interval between the first and the follow-up sample was 49 days. In these longitudinally acquired samples, the nAb titers, as assessed by live-virus NTs, remained stable during the observation period (*P* = 0.0718; Wilcoxon test). Notably, most commercial assays showed a decline in antibody levels over time (Fig. 4; Fig. S1). Such a decline was especially evident for the SARS-CoV-2-NeutraLISA (Euroimmun) (*P* < 0.0001), while antibody levels quantified by the ACE2-RBD neutralization assay (DiaPro) increased (*P* = 0.0001). The median reductions in antibody levels between the first and the second sample measured by the respective assays were as follows: 1.3-fold for the cPass (GenScript), 1.9-fold for the SARS-CoV-2-NeutraLISA (Euroimmun), 1.1-fold for the TECO SARS-CoV-2 neutralization antibody assay (TECOmedical), 1.7-fold for the anti-SARS-CoV-2 QuantiVac ELISA (Euroimmun), and 1.5-fold for the Liaison SARS-CoV-2 TrimericS IgG assay (DiaSorin). The median antibody titers measured by the SARS-CoV-2 ViraChip IgG (Viramed) did not change between the two time points, and the ACE2-RBD neutralization assay (DiaPro) showed a 1.4-fold increase in median antibody levels.

### Antibody assay performance in measuring postvaccination nAb titers.

**FIG 5:**
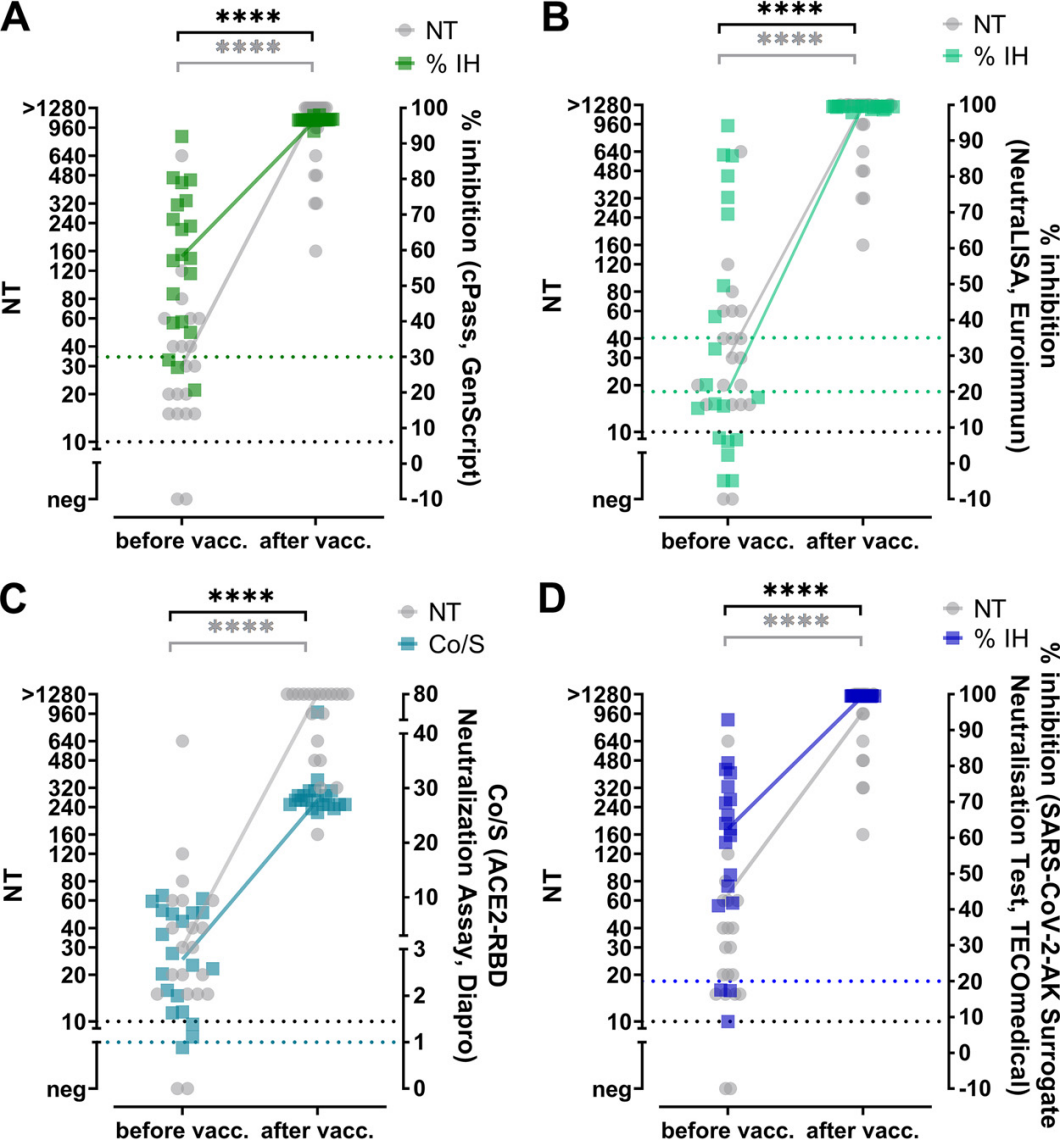
Rise in nAb values between pre- and post-COVID-19 vaccination samples measured by live-virus NT and sVNTs for 20 convalescent individuals. (A) cPass (GenScript); (B) SARS-CoV-2-NeutraLISA (Euroimmun); (C) ACE2-RBD neutralization assay (DiaPro); (D) TECO SARS-CoV-2 neutralization antibody assay (TECOmedical). The dotted lines indicate the cutoffs of the specific assays. Connected lines show medians. Comparison of paired values was performed by the Wilcoxon test. Gray brackets show the results for NT values, and black brackets show those for the respective commercial antibody assay. ****, *P* ≤ 0.0001.

To analyze the performance of the antibody assays to accurately quantify rising nAb levels after COVID-19 vaccination of convalescent individuals, we tested longitudinal samples from 20 convalescent patients who received either one or two vaccine doses. Serum samples were acquired before and after vaccination. Prevaccination samples were obtained at a median of 37 days (range, 19 to 330) after symptom onset and postvaccination samples after a median of 27 days (range, 12 to 63) after a single vaccination (*n* = 18) or 73 days (range, 55 to 91) after two vaccine doses (*n* = 2). Figure 5 and Fig. S2 show that median nAb titers assessed by the live-virus NT significantly increased about 24-fold postvaccination (range, 2- to 85-fold). Similarly, a statistically significant increase in the antibody levels was detected by all commercial assays (*P* < 0.0001; Wilcoxon test). However, the extent of this increase strongly differed among the antibody assays, mainly because the high antibody concentration was out of the detection range of the sVNTs. At the same time, dilution of samples was possible and was performed according to the manufacturers’ instructions in the ELISA, CLIA, and microarray (Fig. 5; Fig. S2).

## DISCUSSION

This study comprehensively compared the ability of seven commercial immunoassays to measure levels of SARS-CoV-2-specific antibodies, which were correlated with specific titers of nAbs quantified by an in-house live-virus NT. A large number of samples from convalescent individuals covering a wide range of nAb titers allowed a statistically conclusive evaluation of the assays’ performances in terms of sensitivity and correlation with the live-virus NT (*P* < 0.0001 for all assays). Furthermore, we identified optimized cutoffs for the commercial immunoassays to assess a defined minimum level of nAbs (cutoff arbitrarily set at a nAb NT titer of ≥80; AUC were >0.87 for all assays). Using this cutoff, we found that the SARS-CoV-2-NeutraLISA by Euroimmun and the cPass by GenScript Biotech were optimal for such an assessment, as indicated by the highest AUC in the respective ROC analyses compared to the other assays.

sVNTs are specifically designed to quantify the antibody-mediated blocking of the first step of virus infection, i.e., the initial binding of the viral RBD to the human receptor ACE2, resulting in a high-grade correlation with titers of nAbs quantified by live-virus NTs (9). In addition, antibody levels measured by the cPass assay displayed the most robust quantitative correlation between the measured antibody levels and titers from the live-virus NT, in line with previous studies (7, 14, 17, 18, 21, 26). Slight differences in the performance of different sVNTs might be explained by differences in assay design (e.g., the cPass assay uses ACE2-coated ELISA plates, while all other assays use RBD binding in the solid phase of the ELISA system) and the processing and structure of the target antigen.

However, our study also clearly demonstrates differences in the sensitivities of commercial sVNTs to detect low levels of nAbs. Notably, of the evaluated sVNTs, the ACE2-RBD neutralization assay by DiaPro and the TECO SARS-CoV-2 neutralization antibody assay by TECOmedical displayed the highest sensitivity in detecting nAbs. These data on the assays’ sensitivities and the observed differences in the optimal detection range for high nAb levels, e.g., in convalescent individuals after COVID-19 vaccination, should help select a specific commercial sVNT for a particular clinical or research application. Such an application could aim either at the highest sensitivity in detecting low levels of nAbs or at the ability to assess higher levels of nAbs at a defined minimum NT titer.

Upon infection with SARS-CoV-2, antibodies against various binding sites of the S protein are produced, and only a particular subset of these antibodies inhibit receptor binding and virus entry, thus providing a neutralizing effect (34). Since the exact amount of antibodies exerting neutralizing capabilities among total antibodies may vary among individuals, the assessment of nAbs by measuring the total concentration of RBD- and S-specific antibodies may be subject to variation. Nonetheless, we show that specific commercial ELISAs, CLIAs, and immunoblots, standardized to BAU per milliliter, were also able to assess live-virus NT titers of ≥80 reliably and were acceptable substitutes for sVNTs with the cutoff values we calculated. In addition, although these cutoff values do not refer to a clinical correlate of protection, similar threshold levels have been shown to correlate with a certain degree of protection against symptomatic SARS-CoV-2 infection with a homologous variant (35).

Another interesting finding was that the longitudinal measurement of nAbs varied depending on the applied antibody assay. Since we observed that the evaluated commercial antibody assays differed in their sensitivities and optimal range for measuring antibody concentrations, these differences may also contribute to variations in the longitudinal assessment of nAbs. Furthermore, the serum antibody composition in longitudinal samples may change for different Ig classes (decline of IgM and IgA and rise of IgG), with IgM and especially IgG yielding the highest neutralizing capabilities, as well as changes in binding avidity over time (36). This may affect different antibody assays to various degrees. Thus, it is essential that when nAb kinetics are assessed in laboratory diagnostics or research, the dependence of the quantified nAb concentrations on the applied antibody assay be considered. A similar impact of the applied commercial antibody assay on the quantitative antibody kinetics has been observed after the COVID-19 vaccination of immunologically naive individuals (11, 26, 27, 30, 33).

In late 2021, the SARS-CoV-2 Omicron variant emerged, and it exhibits a high number of mutations in the S protein, allowing efficient evasion of antibodies induced by previous infections or vaccinations (37, 38). The target antigens of the commercial antibody assays we evaluated in this study are derived from the original wild type of SARS-CoV-2 and have not been adapted to the Omicron variant by the manufacturers so far. Indeed, in an evaluation of commercial assays using samples from convalescent patients after primary Omicron infections, we recently demonstrated reduced sensitivities of S-specific immunoassays (39).

Nonetheless, in the comparative study presented in this paper, we included serum samples only from SARS-CoV-2-convalescent individuals from the first phase of the pandemic before the emergence of the Delta or Omicron variants of concern (VOC) in Austria. Thus, the S- and RBD-specific antibodies in the tested sera matched the target antigens used in the evaluated commercial assays. Accordingly, we used an in-house live-virus NT with the ancestral SARS-CoV-2 strain with the D614G mutation. Nonetheless, we acknowledge that the implications of our data may be limited by the current dominance of the Omicron variant with its immunogenic changes in the S protein. However, our data can still be interpreted as a proof of principle that particular sVNTs, S- and RBD-specific immunoassays, can efficiently assess nAbs when the antigens in the assays match those specific nAbs’ targets.

Importantly, live-virus NTs can specifically detect nAbs against the distinct viral strain used in the assay. Thus, they are still considered a valuable tool when the measurement of SARS-CoV-2-specific antibodies has to be adapted to a newly emerging variant. Therefore, when sVNTs and antibody-binding assays are further used as substitutes for live-virus NTs, they should also be adapted to emerging SARS-CoV-2 variants to more accurately detect nAbs directed explicitly against these variants.

In that regard, our data obtained with multiple commercial SARS-CoV-2-specific sVNTs and anti-spike-IgG antibody assays demonstrate that there may still be considerable assay-specific differences in the sensitivity and the optimal detection range of nAbs, not only in single but also in longitudinal samples. However, they also indicate that the antibody assays evaluated are generally acceptable substitutes for a live-virus NT in assessing quantitative levels of nAbs against SARS-CoV-2.

## MATERIALS AND METHODS

### Samples.

Seven hundred twenty serum samples from 666 nonhospitalized individuals convalescing from coronavirus disease 2019 (COVID-19) (301 [45%] female, 359 [54%] male, and 6 [1%] of unknown sex; median age, 45 years [range, 14 to 97]) (Table 1) after SARS-CoV-2 infection were retested using multiple commercial antibody assays and an in-house live-virus NT. The samples were obtained for routine diagnostic testing (using a commercial antibody assay) between April 2020 and February 2021 (after the first and before the second SARS-CoV-2 wave in Austria). Residual sample material from routine diagnostics was anonymized and integrated into a biobank using a protocol approved by the local ethics committee (EK 1035/2016 and EK 1513/2016). Infection with SARS-CoV-2 was confirmed by RT-PCR from nasopharyngeal swabs (*n* = 603) or by the in-house live-virus NT (*n* = 63).

Because diagnosis occurred between March 2020 and January 2021, infection with SARS-CoV-2 variants other than the ancestral D614G or Alpha strains, which circulated in Austria during this period, could be excluded (40). Clinical information was obtained and documented on the laboratory assignment sheet by the treating physicians or blood donation centers. Twenty convalescent vaccinees with documentation of one or two doses of a monovalent mRNA- (BioNTech Pfizer or Moderna) or vector-based (Oxford AstraZeneca or Johnson & Johnson) COVID-19 vaccine following SARS-CoV-2 infection were included. The absence of vaccination was specifically documented for all other individuals, or blood collection occurred before SARS-CoV-2 vaccination became available in Austria.

Since all individuals consented to the initial SARS-CoV-2-specific antibody testing at the Center for Virology, and only anonymized samples were retested for this study, the local ethics committee concluded that no written consent was required for these analyses (EK 2156/2019).

Serum samples from 234 healthy individuals (112 [48%] female, 122 [52%] male; median age, 45 years [range, 14 to 91]) were used as specificity control samples (defined as true negatives for specificity analyses). These samples were initially sent to the Center for Virology before the SARS-CoV-2 pandemic in June 2019 to determine measles virus-, mumps virus- and rubella virus-specific antibodies postvaccination. Samples from these controls were age and sex matched to samples from COVID-19-convalescent individuals.

### Commercial antibody tests.

Nine hundred fifty-four serum samples from COVID-19-convalescent individuals (*n* = 720) and prepandemic controls (*n* = 234) were retested with the following seven commercial antibody assays (four sVNTs, one ELISA, one CLIA, and one protein microarray): cPass (GenScript Biotech, Piscataway, NJ, USA), SARS-CoV-2-NeutraLISA (Euroimmun, Lübeck, Germany), ACE2-RBD neutralization assay (DiaPro Diagnostic Bioprobes, Sesto San Giovanni, Italy), TECO SARS-CoV-2 neutralization antibody assay (TECOmedical, Sissach, Switzerland), anti-SARS-CoV-2 QuantiVac ELISA (Euroimmun, Lübeck, Germany), Liaison SARS-CoV-2 TrimericS IgG CLIA (DiaSorin, Saluggia, Italy), and SARS-CoV-2 ViraChip IgG microarray (Viramed Biotech, Planegg, Germany).

Detailed information on all evaluated assays, including the respective target antigens, the base principles of the tests, cutoff values, and covered immunoglobulin classes, are shown in Table S1. All tests were performed following the manufacturers’ instructions, strictly adhering to the respective protocol, and performing only dilution steps recommended by the manufacturers.

### Live-virus NT.

An in-house live-virus NT was performed following a previously described protocol to detect neutralizing antibodies in serum samples (41). In brief, 50 to 100 50% tissue culture infective doses (TCID_50_) of an ancestral SARS-CoV-2 strain with the D614G mutation (GISAID/EPI_ISL_438123/hCoV-19/Austria/CeMM0360/2020) was incubated with 2-fold serial dilutions of heat-inactivated patient serum or plasma (starting at a dilution of 1:10) for 1 h at 37°C. The mixture was then applied to Vero E6 (ECACC 85020206) cell monolayers, and incubation was carried out for 3 days. Neutralization titers were expressed as the reciprocal of the serum dilution required for protection against virus-induced cytopathic effects. A neutralization titer of ≥10 was considered positive. Test specificity was 100% as assessed with prepandemic controls (41).

### Statistical analyses.

Demographic data are depicted in tabular form. Fisher’s exact test was applied to calculate the sensitivities and specificities of the commercial antibody assays.

The antibody assays’ detection rates were first analyzed with samples from individuals with reverse transcription-PCR (RT-PCR)-confirmed SARS-CoV-2 infection to assess their ability to detect past SARS-CoV-2 infection by any level of specific antibodies (true positives: RT-PCR-confirmed infections). Then, the assays’ detection rates were compared in samples that tested positive in the live-virus NT to assess their ability to detect nAbs of any concentration (true positives: samples positive by the live-virus NT).

Log transformation of data was performed for correlation analyses, but the Shapiro-Wilk test revealed that the values were still not normally distributed. Spearman’s rho test was thus used for correlation analyses for nonparametric data (correlation between live-virus NT titers with quantitative results from the commercial antibody assays), and the coefficient of determination (*R*^2^) was determined to assess variability. Only one sample per individual was used for these analyses in cases where one individual provided multiple samples. The earlier sample was analyzed to calculate sensitivity. In vaccinated convalescent patients, the (latest) sample obtained after the vaccination was used for the correlation analyses.

ROC curve analyses were performed with untransformed data to calculate sensitivities and specificities of individual assays at a live-virus NT titer cutoff of 80. This cutoff was chosen based on previous observations in our laboratory, which showed that a minimum titer of 80 was required to maintain at least minimal neutralization capacity against heterologous SARS-CoV-2 strains (e.g., the Delta variant).

To assess the antibody assays’ ability to quantify the decrease of SARS-CoV-2-specific antibodies in relation to the live-virus NT and the increase from pre- to postvaccination titers, median fold reduction or increase were calculated intraindividually. Test results for these calculations are graphically shown as individual values plus medians. In addition, the Wilcoxon test was performed to calculate differences in variances for the comparison of paired groups. GraphPad Prism (version 9.2.0; GraphPad Software, San Diego, CA, USA) was used for statistical analysis. A *P* value of <0.05 was considered statistically significant.
