# Supplementary material for: Comprehensive Comparison of Seven SARS-CoV-2-Specific Surrogate Virus Neutralization and Anti-Spike IgG Antibody Assays Using a Live-Virus Neutralization Assay as a Reference
Source: Microbiol Spectr. 2023 Jan 9;11(1):e02314-22. doi: 10.1128/spectrum.02314-22 (PMC9927416; doi:10.1128/spectrum.02314-22)
Supplement: Supplemental file 1 — Fig. S1 and S2 and Tables S1 and S2. Download spectrum.02314-22-s0001.pdf, PDF file, 0.8 MB [file spectrum.02314-22-s0001.pdf]

# Supplementary Information

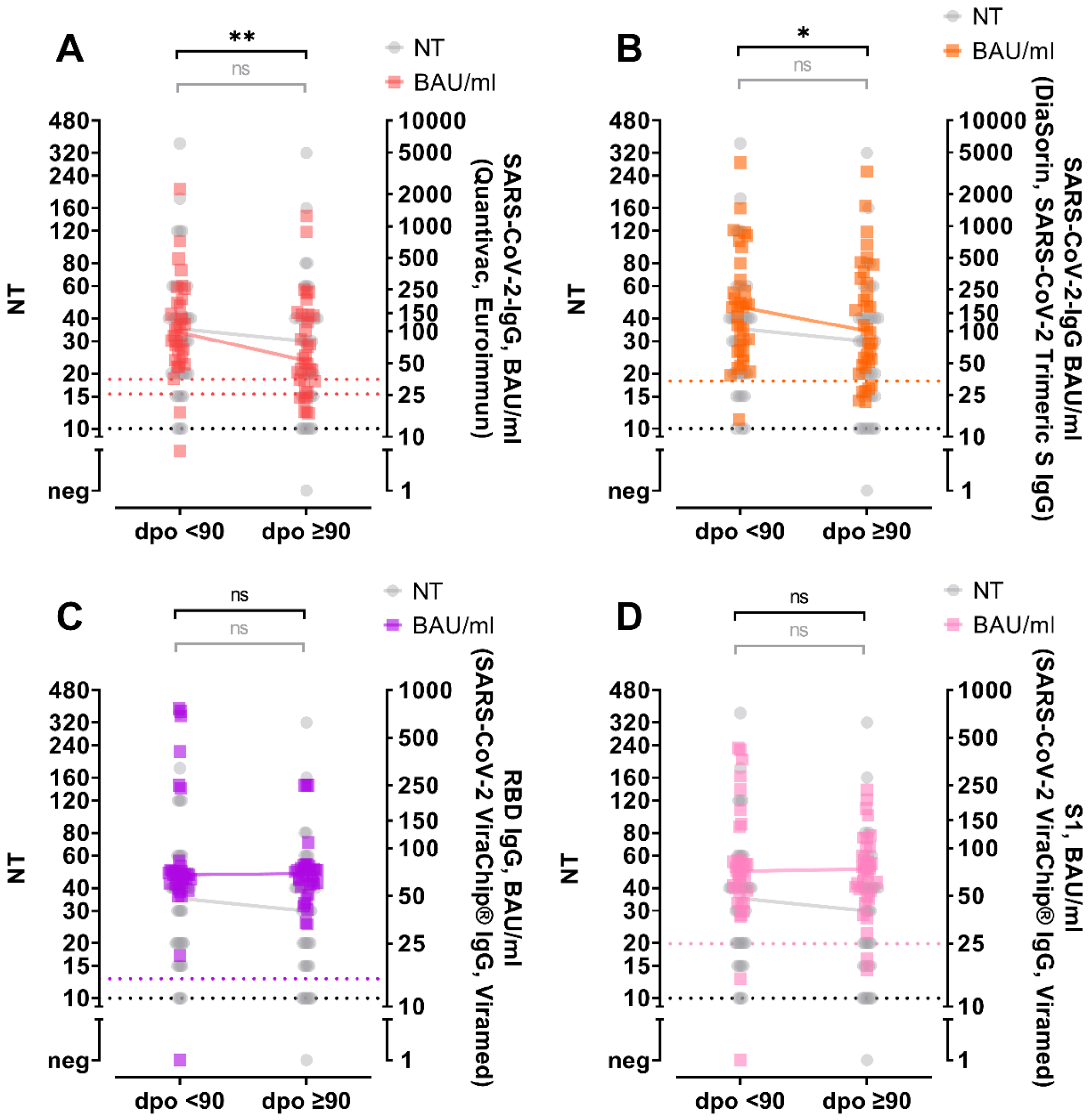


**Supplementary Figure S1: Kinetics of nAbs measured by live-virus NT and conventional binding assays.** Samples of 34 convalescent individuals with the first sample <90 days post onset and an additional sample ≥90 days post onset were tested with NT and all sVNTs. (A) Anti-SARS-CoV-2 QuantiVac ELISA (Euroimmun), (B) LIAISON® SARS-CoV-2 TrimericS IgG CLIA (DiaSorin,), (C) SARS-CoV-2 ViraChip® IgG microarray (Viramed Biotech) RBD and (D) S1 directed IgG. The dotted lines indicate the cut-offs of the specific assays. Connected lines show medians. Comparison of paired values was performed by

Wilcoxon test. Grey brackets show the results for NT values, and black brackets for the respective commercial antibody assay. Asterisks indicate statistical significance: (*) = P ≤ 0.05, (**) = P ≤ 0.01, (***) = P ≤ 0.001, (****) = P ≤ 0.0001.

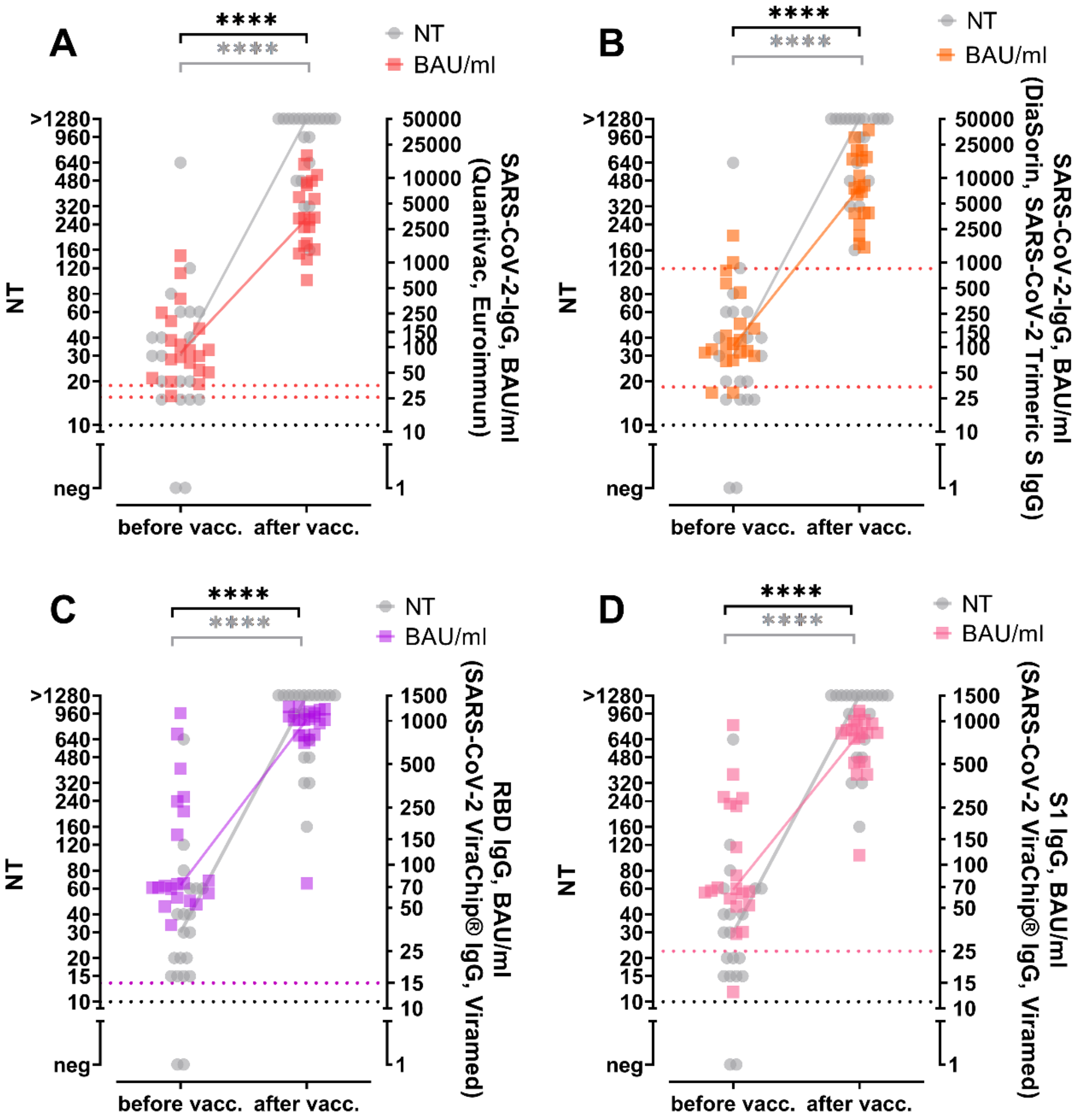


**Supplementary Figure S2: Rise in nAb values between pre- and post-COVID-19 vaccination samples measured by live-virus NT and sVNTs in 20 convalescent individuals.** (A) Anti-SARS-CoV-2 QuantiVac ELISA (Euroimmun), (B) LIAISON® SARS-CoV-2 TrimericS IgG CLIA (DiaSorin,), (C) SARS-CoV-2 ViraChip® IgG microarray (Viramed Biotech) RBD and (D) S1 directed IgG. The dotted lines indicate the

cut-offs of the specific assays. Connected lines show medians. Comparison of paired values was performed by Wilcoxon test. Grey brackets show the results for NT values, and black brackets for the respective commercial antibody assay. Asterisks indicate statistical significance: (*) = $P \leq 0.05$, (**) = $P \leq 0.01$, (***) = $P \leq 0.001$, (****) = $P \leq 0.0001$.

**Supplementary Table S1: SARS-CoV-2-specific immunoassays**

| Test | Manufacturer | Principle | Target Antigen | Immunglobulin class | Unit | Cut-off | | |
|---|---|---|---|---|---|---|---|---|
| | | | | | | negative | borderline | positive |
| **cPass™ SARS-CoV-2 Neutralization Antibody Detection Kit** | GenScript, New Jersey, US | ELISA | RBD of S1 | IgG, IgM, IgA | % inhibition | < 30 | - | ≥ 30 |
| **SARS-CoV-2-NeutraLISA** | Euroimmun, Lübeck, Germany | ELISA | RBD of S1 | IgG, IgM, IgA | % inhibition | < 20 | ≥ 20 to < 35 | ≥ 35 |
| **ACE2-RBD Neutralization Assay – ELISA** | DiaPro, Sesto San Giovanni, Italy | ELISA | RBD of S1 | IgG, IgM, IgA | Co/S | ≤1 | - | >1 |
| **TECO SARS-CoV-2-AK Surrogate Neutralisation Test** | TECOmedical, Sissach, Switzerland | ELISA | RBD of S1 | IgG, IgM, IgA | % inhibition | < 20 | - | ≥ 20 |
| **Anti-SARS-CoV-2-QuantiVac-ELISA (IgG)** | Euroimmun, Lübeck, Germany | ELISA | S1 | IgG | BAU/ml | < 25.6 | ≥ 25.6 - < 35.2 | ≥ 35.2 |
| **LIAISON® SARS-CoV-2 TrimericS IgG assay** | Diasorin, Saluggia, Italy | CLIA | S1 | IgG | BAU/ml | < 33.8 | - | ≥ 33.8 |
| **SARS-CoV-2 ViraChip® IgG assay** | Viramed, Planegg, Germany | Microarray | S1 | IgG | BAU/ml | <25 | - | ≥ 25 |
| | | | RBD of S1 | IgG | BAU/ml | <15 | - | ≥ 15 |

**Supplementary Table S2: Specificity of the evaluated immunoassays**

| | **Negative controls (N=234)** | |
|---|---|---|
| | **Specificity in % (95% CI)** | **n/N** |
| **cPass™ SARS-CoV-2 Neutralization Antibody Detection Kit (GenScript)** | | |
| „negative“ (<30% IH) | 100.00 (98.38-100.00) | 234/234 |
| **SARS-CoV-2-NeutraLISA (Euroimmun)** | | |
| "negative" (<20% IH) | 97.86 (95.10-99.08) | 229/234 |
| **ACE2-RBD Neutralization Assay (Diapro)** | | |
| “negative” (<1 Co/S) | 100.00 (98.38-100.00) | 234/234 |
| **SARS-CoV-2-AK Surrogate Neutralisation Test (TECOmedical)** | | |
| „negative“ (<20% IH) | 100.00 (98.38-100.00) | 234/234 |
| **Anti-SARS-CoV-2-QuantiVac-ELISA (IgG) (Euroimmun)** | | |
| "negative" (<35.2 BAU/ml) | 99.15 (96.94-99.85) | 232/234 |
| **LIAISON® SARS-CoV-2 TrimericS IgG (Diasorin)** | | |
| "negative" (<33.8 BAU/ml) | 100.00 (98.38-100.00) | 234/234 |
| **SARS-CoV-2 ViraChip® IgG assay (Viramed)** | | |
| **RBD IgG** | | |
| “negative” (<15 BAU/ml) | 98.29 (95.96-99.33) | 230/234 |
| **S1 IgG** | | |
| “negative” (<25 BAU/ml) | 99.57 (97.62-99.98) | 233/234 |
